# VHL‐Mediated SYT11 Degradation Suppresses Gastric Cancer Cell Growth and Invasion Through Downregulation of SPINK1

**DOI:** 10.1111/jcmm.70658

**Published:** 2025-06-27

**Authors:** Ji Su Yu, Mi‐Aie Hwang, Misun Won, Joo‐Young Im, Dae‐Hyuk Kweon, Yun Gyu Park, Bo‐Kyung Kim

**Affiliations:** ^1^ Genomic Medicine Research Center Korea Research Institute of Bioscience and Biotechnology Daejeon Korea; ^2^ Department of Biochemistry and Molecular Biology Korea University College of Medicine Seoul Korea; ^3^ Department of Integrative Biotechnology College of Biotechnology and Bioengineering, Sungkyunkwan University Suwon Korea; ^4^ Biotherapeutics Translational Research Center Korea Research Institute of Bioscience and Biotechnology Daejeon Korea; ^5^ Korean Institute of Molecular Medicine and Nutrition Korea University College of Medicine Seoul Korea; ^6^ KRIBB School of Bioscience University of Science and Technology Daejeon Korea

**Keywords:** gastric cancer, SPINK1, SYT11, ubiquitination, VHL

## Abstract

The ubiquitin‐proteasome system is a post‐translational modification pathway that plays a critical role in regulating cell survival and death. E3 ubiquitin ligases are key tumour regulators and potential therapeutic targets in gastric cancer. This study investigates whether von Hippel–Lindau (VHL), an E3 ligase, regulates the stability of synaptotagmin 11 (SYT11) protein in gastric cancer cells. VHL overexpression decreased SYT11 protein expression without affecting SYT11 mRNA expression. Notably, VHL overexpression decreased the half‐life of SYT11 protein, and MG132, a proteasome inhibitor, reversed SYT11 degradation by VHL. Immunoprecipitation confirmed the binding of SYT11 to VHL, and VHL knockdown resulted in reduced SYT11 ubiquitination and degradation. Transcriptome sequencing revealed the downregulation of serine peptidase inhibitor kazal type 1 (SPINK1) by VHL and its upregulation by SYT11. VHL downregulated the expression of SYT11, which subsequently led to the inhibition of SPINK1 expression. Furthermore, SPINK1 knockdown inhibited the growth and invasion of gastric cancer cells, mirroring VHL overexpression effects. The inhibition of growth and invasion in MKN1 and SNU484 cells by VHL was rescued by the overexpression of SYT11 and SPINK1. These findings demonstrate that the proteasome‐dependent degradation of SYT11 by VHL and the subsequent reduction in SPINK1 expression inhibit gastric cancer cell growth and invasion.

## 1 Introduction

Ubiquitin, a 76‐amino‐acid protein, is universally expressed in eukaryotes. Ubiquitination is a form of post‐translational modification involving the covalent conjugation of ubiquitin to target proteins via lysine residues. This process is mediated by a sequential, ATP‐dependent enzymatic cascade, comprising an E1 activating enzyme, an E2 conjugating enzyme, and an E3 ligase. Ubiquitination plays an important role in cell survival and death. Various ubiquitin systems have been implicated in cell death and autophagy pathways and may even crosstalk with each other [1]. The X‐linked inhibitor of apoptosis protein (XIAP), an ubiquitin ligase, promotes autophagosome‐lysosome fusion but inhibits apoptosis through Caspase3/7 [2]. TNF receptor‐associated factor 6 (TRAF6) ubiquitinates unc‐51‐like autophagy‐activating kinases 1 (ULK1) but inhibits extrinsic apoptosis [3].

Among the over 650 E3 ligases reported in humans, the really interesting new gene (RING) and homologous to the E6‐AP Carboxyl Terminus (HECT) family members have garnered attention for their roles in gastric cancer. Specifically, these E3 ligases influence the proliferation and infiltration of gastric cancer cells and impact prognosis [4]. For example, ring finger protein 38 (RNF 38) induces polyubiquitination and degradation of tyrosine‐protein phosphatase SHP‐1, promoting gastric cancer cell proliferation [5]. Conversely, E3 Ubiquitin‐protein ligase UBR5 downregulates Gastrokine 1, a gastric cancer suppressor, thereby promoting cell growth [6]. Notably, UBR5 overexpression was shown to be associated with poor overall and disease‐free survival.

Loss of function of the tumour suppressor gene VHL is a known cause of clear cell renal cell carcinoma (ccRCC) [7]. Approximately 70% of ccRCC cases result from the inactivation of the VHL gene through mutation, deletion, or promoter methylation. The protein product of the VHL, pVHL, serves as the substrate recognition unit of an E3 ubiquitin ligase complex and targets hypoxia‐inducible factor (HIF) for ubiquitination and proteasomal degradation. Furthermore, pVHL regulates cancer cell proliferation by targeting epidermal growth factor receptor (EGFR), retinol binding protein 1 (RBP1), and protein kinase C (PKC) [8]. Recent findings demonstrate the potential of VHL in targeting gastric cancer. Specifically, a VHL‐recruiting vascular endothelial growth factor receptor 2 (VEGFR‐2) PROTAC (proteolysis‐targeting chimera) was shown to degrade VEGFR‐2 protein, inducing apoptosis in gastric cancer cells [9]. Additionally, VHL‐mediated downregulation of NIMA‐associated kinase 8 (NEK8) inhibited gastric cancer cell proliferation and migration [10]. The VHL isoform with short 3′‐UTR inhibits cell growth and enhances apoptosis in gastric cancer cells [11]. Despite these promising results, the molecular substrates and downstream mechanisms of VHL in gastric cancer remain incompletely understood. Interestingly, although VHL has traditionally been known for its role in hypoxia signalling, recent studies have expanded its substrate scope to include proteins outside the HIF pathway [12, 13].

SYT11 is a highly expressed gene in the stem‐like molecule subtype of gastric cancer and is associated with poor prognosis. In our previous study, SYT11 was found to promote gastric cancer development through the MAP kinase (MKK7)‐c‐Jun N‐terminal kinases (JNK) pathway [14]. SYT11 has also been reported to regulate Impad‐1‐mediated metastasis in lung cancer [15]. Notably, SYT11 undergoes ubiquitination‐mediated degradation, a process facilitated by Parkin. In the absence of Parkin, SYT11 accumulates, leading to Parkinson's disease‐like neurotoxicity [16]. Loss of the ATP13A2 gene (also known as PARK9) results in abnormal aggregation of α‐synuclein in Parkinson's disease [17]. ATP13A2 gene depletion in HeLa cells induced SYT11 degradation and autophagy blockage [18].

SPINK1 plays an important role in cancer development and progression, with context‐dependent functions [19, 20, 21]. It promotes cell growth and invasion in lung and colon cancers [22, 23]. As a secreted protease inhibitor of pancreatic trypsin [24], SPINK1 is frequently overexpressed in pancreatic cancer, contributing to its development and progression [25]. Although the role of SPINK1 in gastric cancer remains largely unexplored, elevated serum SPINK1 levels have been linked to advanced‐stage gastric cancer [26].

VHL and SYT11 contribute to gastric cancer progression via distinct pathways, but both are involved in the ubiquitin–proteasome system, suggesting possible convergence in post‐translational control. This overlap implies potential crosstalk between their signalling networks, especially in cancer‐related contexts involving autophagy or vesicular trafficking [18, 27, 28, 29]. In our preliminary study, Parkin was not detected in several gastric cancer cell lines, while VHL was expressed and found to directly interact with SYT11. These findings prompted us to investigate whether VHL regulates SYT11, a potential oncogenic driver in gastric cancer.

In this study, we have uncovered a novel mechanism by which VHL decreases SYT11 protein stability, thereby downregulating SPINK1 expression and inhibiting the growth and invasion of gastric cancer cells.

## 2 Materials and Methods

### 2.1 Cell Culture

Human gastric cancer cell lines (AGS, KATOIII, Hs746T, SNU484, MKN1, and SKGT4) were cultured in RPMI‐1640 medium (Welgene, Gyeongsan‐si, Korea) supplemented with 10% fetal bovine serum (Welgene, Korea) and 1% penicillin/streptomycin (Gibco, Grand Island, NY, USA). All cell lines were maintained in a humidified atmosphere with 5% CO_2_ at 37°C.

### 2.2 Reagents

Cycloheximide and MG132 were purchased from Sigma‐Aldrich (St. Louis, MO, USA). Lipofectamine RNAiMAX reagent and TurboFect transfection reagent were purchased from Invitrogen (Carlsbad, CA, USA) and Thermo Fisher Scientific (Waltham, MA, USA), respectively. siVHL (7428‐1) and siSPINK1 (6690‐1) were sourced from Bioneer (Daejeon, Korea). The siRNA sequences were siScramble (5′‐CCUACGCCACCAAUUUCGU (dTdT)‐3′) and siSYT11 (5′‐GCAGAAAGCGCAUUGCCAA (dTdT)‐3′).

### 2.3 Plasmids

Plasmids pCMV3, pCMV3‐HA‐SYT11, pCMV3‐GFP‐SYT11, pCMV3‐GFP‐SPINK1, and pCMV3‐HA‐Parkin were purchased from Sino Biological (Wayne, PA, USA). Mutant constructs (K74A and K217A) of pCMV3‐HA‐SYT11 were generated using the EZ Change Site‐Directed Mutagenesis Kit (Enzynomics, Daejeon, Korea). The sequences of the primer pairs were used as follows: SYT11‐K74A (5′‐CATCATCAAAGTGCGGAGAGACAAAGA‐3′ and 5′‐GCCTTCTTGTTGCTGAGGGTCTCTGGG‐3′), and SYT11‐K217A (5′‐CACCCTGGACCCTGTGTTTGACGAGAC‐3′ and 5′‐GCCCGCAGCACTCTGGTCTTCACCCGA‐3′). The VHL cDNA clones (1‐213 amino acids) were obtained from the Korea Human Gene Bank (Daejeon, Korea). The VHL coding region was amplified by PCR and then cloned into the pcDNA3‐2xMyc plasmid between the HindIII/EcoRV sites, yielding pcDNA3‐2xMyc‐VHL. The coding region of ubiquitin (1‐76 amino acids) was PCR‐amplified from the HEK293 cDNA and inserted into the pcDNA3.1‐HA plasmid between the EcoRI/XhoI sites.

### 2.4 Immunoprecipitation and Western Blotting

Cells were lysed in RIPA buffer (Millipore, Billerica, MA, USA) supplemented with a protease inhibitor cocktail (Roche, Basel, Switzerland). Protein concentrations were quantified using the BCA Protein Assay Kit (Thermo Fisher Scientific, Waltham, MA, USA). For immunoprecipitation (IP), cell lysates were incubated with antibody and Protein A/G Plus agarose (Santa Cruz Biotechnology, Dallas, Texas, USA) at 4°C with rotation. After centrifugation, agarose pellets were washed with IP buffer. Proteins were identified using the following antibodies: anti‐SYT11 (Abcam, Cambridge, UK), anti‐SPINK1 (Abcam), anti‐HA (Santa Cruz Biotechnology, Dallas, TX, USA), anti‐GFP (Santa Cruz Biotechnology), anti‐β‐actin (Santa Cruz Biotechnology), anti‐VHL (Cell signalling Technology, Danvers, MA, USA), anti‐Myc (Cell signalling Technology), anti‐β‐tubulin (Cell signalling Technology), and anti‐GAPDH (Ab Frontier, San Jose, CA, USA).

### 2.5 Reverse Transcription‐PCR (RT‐PCR) and Quantitative RT‐PCR (qRT‐PCR)

Total RNA isolation and cDNA synthesis were performed as previously described [30]. RNA was extracted using Trizol reagent (Invitrogen, Carlsbad, CA, USA), followed by cDNA synthesis with TOPscript RT DryMIX (Enzynomics, Daejeon, Korea). RT‐PCR was then conducted using AccuPower Taq PCR Master Mix (Bioneer, Daejeon, Korea). qRT‐PCR was performed using the SYBR Green qPCR Master Mix (BIOFACT, Daejeon, Korea) on a CFX Duet Real‐Time PCR System (Bio‐Rad, Hercules, CA, USA). The following primers were used: RPL13A (5′‐CATCGTGGCTAAACAGGTAC‐3′ and 5′‐GCACGACCTTGAGGGCAGC‐3′), VHL (5′‐GAGTCCGGCCCGGAAGAGTCGC‐3′ and 5′‐CATCGTGTGTCCCTGCATCTCT‐3′). The SYT11 (P281379) and SPINK1 (P325987) primers were purchased from Bioneer (Daejeon, Korea).

### 2.6 Cell Proliferation Assay

Cell proliferation was assessed using a live cell imaging system (CellPro, NanoSystem, Daejeon, Korea), which measures cell confluence. Each sample was evaluated in triplicate (three wells), with five images analysed per well.

### 2.7 Cell Viability and Invasion Assay

Cell viability was evaluated using the Sulforhodamine B (SRB) assay, as previously described [31]. For invasion assays, 24‐well inserts with 0.8 μm pore membranes (BD Biosciences, Franklin Lakes, NJ, USA) were used, as previously described [32]. Cells were seeded into the upper chamber, coated with Matrigel (BD Biosciences), while the lower compartments contained medium. To analyse adherent or invasive cells, the cells were fixed with 10% formalin and stained with 0.4% SRB (Sigma‐Aldrich, St. Louis, MO, USA). Subsequently, the cells were washed with 0.01 M acetic acid. The protein‐bound dye was dissolved in 10 mM Tris, and its optical density was measured using a Synergy HTX Multi‐Mode Reader (BioTek, Winooski, VT, USA).

### 2.8 Transcriptome Sequencing

Total RNA extracted from MKN1 cells was used to construct a library using the TruSeq Stranded mRNA LT Sample Prep kit. Sequencing was conducted on the Illumina platform (Macrogen, Seoul, Korea). The resulting dataset is available in the Korea BioData Station (K‐BDS) under registration number KAP240808.

### 2.9 Statistical Analyses

Data are expressed as mean ± standard deviation and between‐group differences were assessed using the Student's *t*‐test. Statistical analyses were conducted using Prism software (GraphPad, Boston, MA, USA).

## 3 Results

### 3.1 VHL Inhibits SYT11 Protein Expression in Gastric Cancer Cells

**FIGURE 1:**
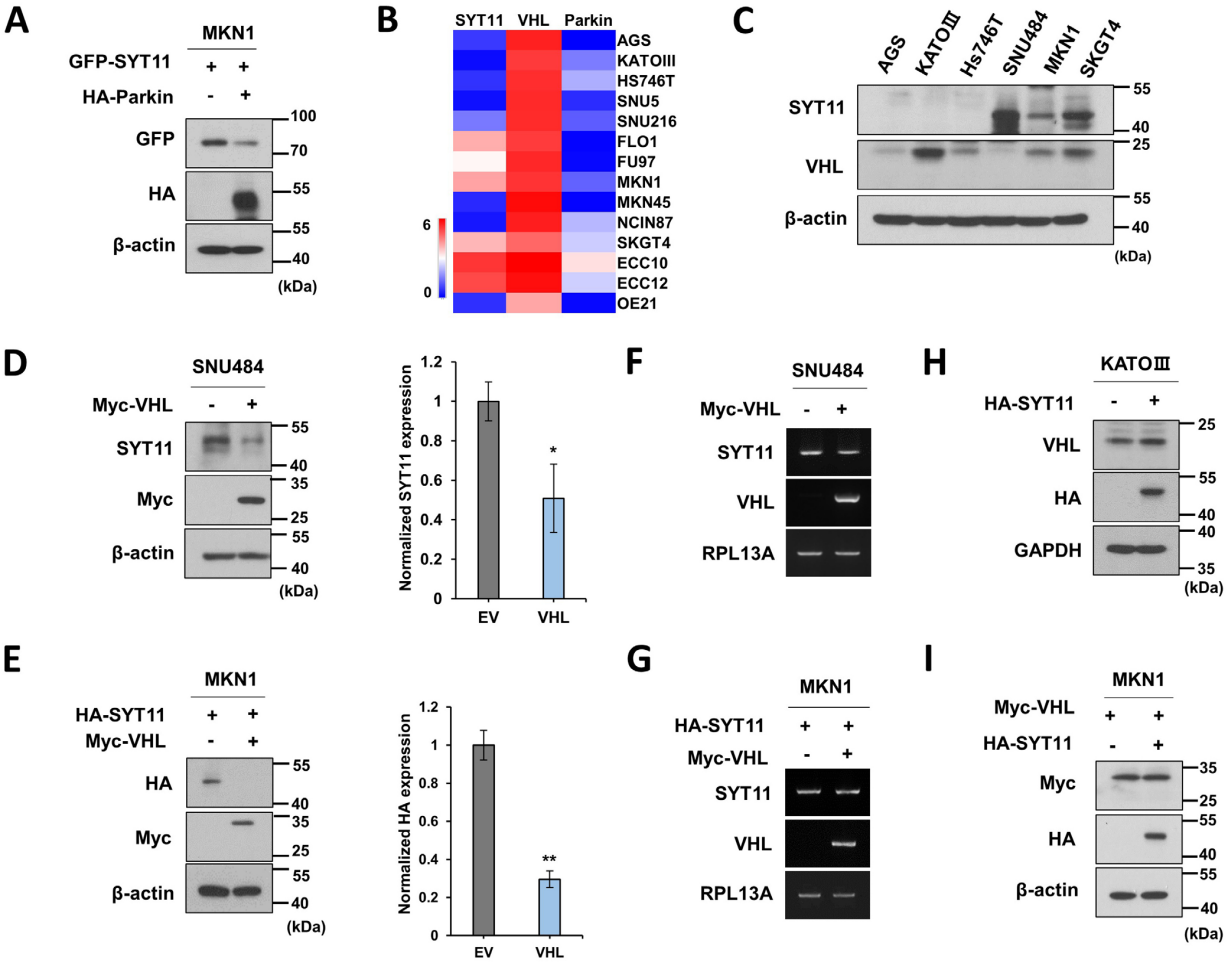
VHL inhibits SYT11 protein expression. (A) Western blot analysis of MKN1 cells transfected with GFP‐SYT11 and HA‐Parkin using Turbofect for 48 h. (B) Comparative mRNA expression analysis of SYT11, VHL, and Parkin in gastric cancer cell lines (depmap.org). (C) Western blot analysis of SYT11 and VHL protein expression in gastric cancer cell lines. (D, E) Western blot analysis of SNU484 cells transfected with Myc‐VHL (D) and MKN1 cells transfected with Myc‐VHL and HA‐SYT11 (E). Band intensity was analysed using ImageJ software (*n* = 3). (F, G) RT‐PCR analysis of the effect of VHL on SYT11 mRNA expression. (H, I) Western blot analysis of KATOIII cells transfected with HA‐SYT11 (H) and MKN1 cells transfected with Myc‐VHL and HA‐SYT11 (I). **p ≤ 0.01; *p ≤ 0.05 (Student's *t*‐test).

Consistent with previous studies, SYT11 expression in gastric cancer cell line MKN1 was downregulated by Parkin (Figure 1A). We compared the expression levels of Parkin and another E3 ligase, VHL, in gastric cancer cell lines. Analysis of the Cancer Dependency Map (DepMap) showed low Parkin mRNA expression in most gastric cancer cell lines, while VHL mRNA expression was elevated (Figure 1B). Western blotting confirmed VHL protein expression in gastric cancer cell lines (Figure 1C), whereas Parkin was undetectable (data not shown).

Next, we investigated the effect of VHL on SYT11 expression in gastric cancer cells. VHL overexpression in SNU484 cells reduced endogenous SYT11 expression (Figure 1D). Similarly, transient cotransfection of SYT11 and VHL into MKN1 cells resulted in VHL‐mediated inhibition of exogenous SYT11 expression (Figure 1E). Notably, VHL overexpression did not alter SYT11 mRNA levels (Figure 1F,G). Conversely, SYT11 overexpression in KATOIII or MKN1 cells, which were transiently transfected with VHL, did not affect VHL protein expression (Figure 1H,I). These findings demonstrate that VHL specifically suppresses SYT11 protein expression without affecting SYT11 mRNA levels.

### 3.2 VHL Regulates the SYT11 Protein Stability

**FIGURE 2:**
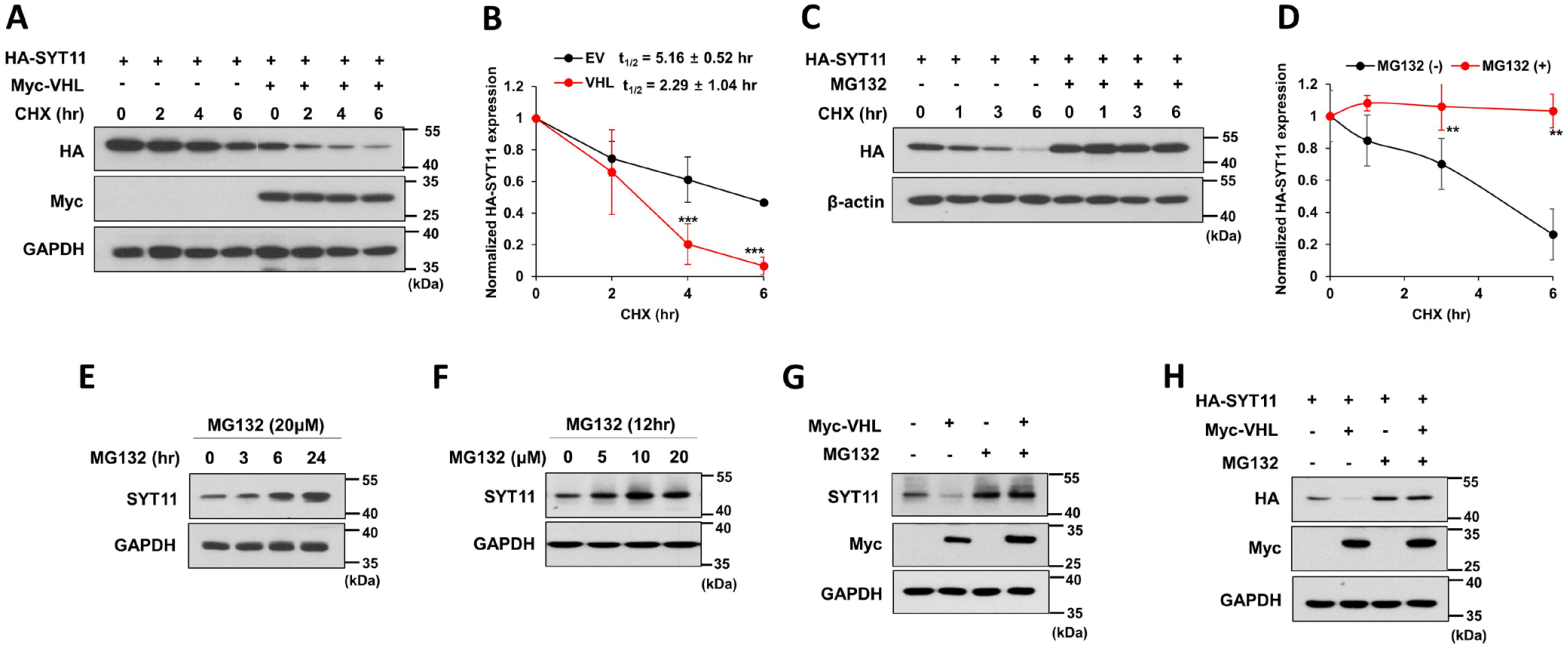
VHL‐mediated SYT11 reduction is proteasome‐dependent. (A, B) MKN1 cells were transfected with HA‐SYT11 and Myc‐VHL for 48 h, then incubated with 100 μg/mL cycloheximide (CHX) for the indicated durations. The expression of HA‐SYT11 was normalised to GAPDH. The graph shows the mean value from three independent experiments. *t*_1/2_ represents the half‐life of the protein. (C, D) MKN1 cells transfected with HA‐SYT11 were pretreated with 20 μM MG132 for 24 h and then treated with CHX. HA‐SYT11 expression was normalised to β‐Actin. The graph shows the mean value from three independent experiments. (E, F) SNU484 cells were treated with MG132 for the indicated durations or the indicated concentrations. (G, H) Western blot analysis was performed on SNU484 cells transfected with Myc‐VHL (G) and MKN1 cells transfected with HA‐SYT11 and Myc‐VHL (H) for 24 h, followed by treatment with or without 10 μM MG132 for 12 h. ***p ≤ 0.001; **p ≤ 0.01 (Student's *t*‐test).

To investigate the mechanism underlying VHL‐mediated regulation of SYT11, we treated MKN1 cells with cycloheximide (CHX), an inhibitor of protein synthesis. Under CHX treatment, VHL overexpression decreased the half‐life of SYT11 protein (Figure 2A,B), indicating that SYT11 is regulated post‐translationally but not translationally. To examine the role of ubiquitination‐mediated degradation, we treated the cells with proteasome inhibitor MG132. MG132 inhibited the time‐dependent degradation of SYT11 protein in cells treated with CHX (Figure 2C,D) and increased endogenous SYT11 expression in a time‐ and dose‐dependent manner (Figure 2E,F). Notably, MG132 also rescued SYT11 expression reduced by VHL overexpression (Figure 2G,H). These results demonstrate that VHL regulates SYT11 expression via proteasome‐dependent degradation.

### 3.3 VHL Binds to SYT11 and Induces Its Ubiquitination

**FIGURE 3:**
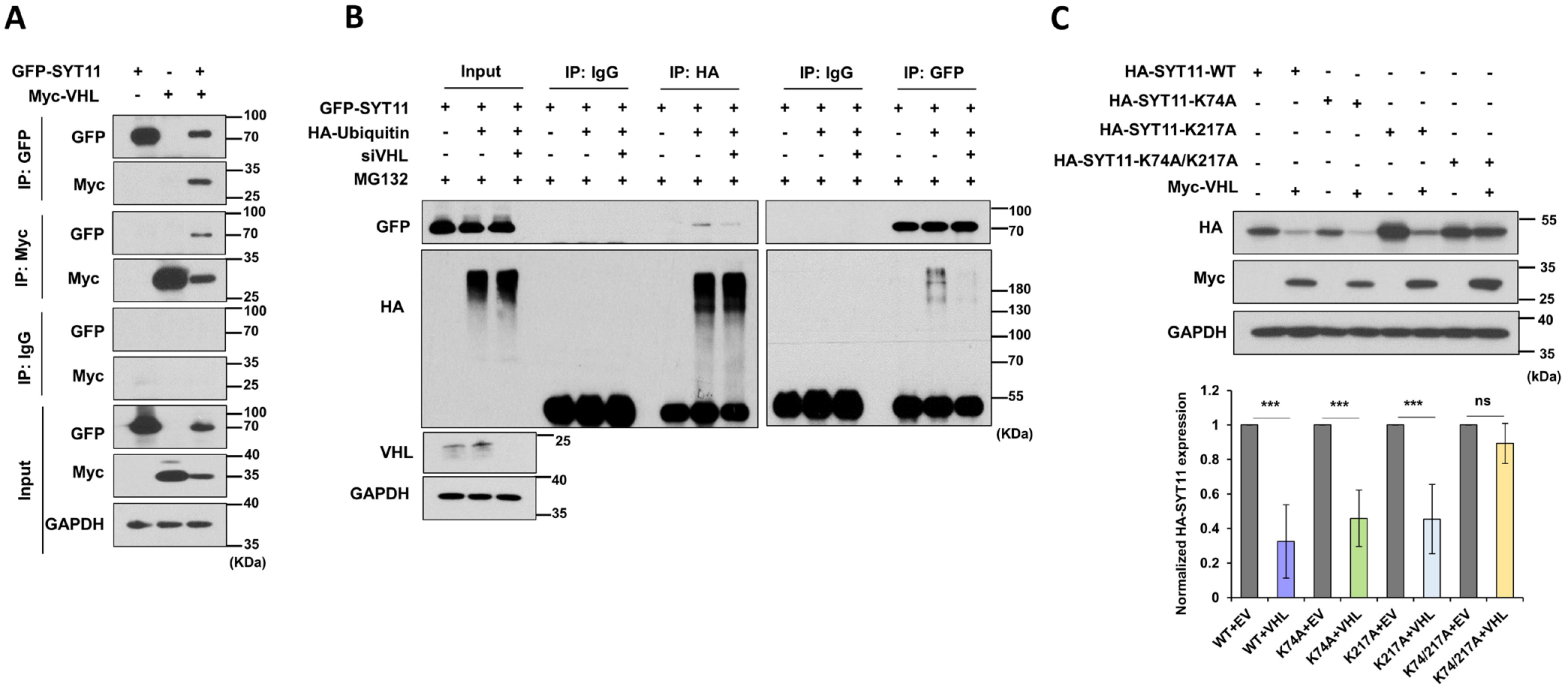
VHL mediates SYT11 ubiquitination. (A) Lysates of MKN1 cells transfected with GFP‐SYT11 and Myc‐VHL were immunoprecipitated with anti‐Myc or anti‐GFP antibodies and immunodetected with the indicated antibodies. (B) MKN1 cells treated with 40 μM siVHL were transfected with GFP‐SYT11 or HA‐Ubiquitin and then treated with MG132 for 12 h. Immunoprecipitation was performed with anti‐HA or anti‐GFP antibodies, followed by immunodetection with the indicated antibodies. (C) MKN1 cells were transfected with HA‐SYT11, HA‐SYT11‐K74A, HA‐SYT11‐K217A, HA‐SYT11‐K74A/K217A, and Myc‐VHL for 48 h using Turbofect. Band intensity was quantified using ImageJ software (*n* = 3). ns, no significance; ***p ≤ 0.001 (Student's *t*‐test).

To confirm the direct interaction between SYT11 and VHL, we performed immunoprecipitation in MKN1 cells transfected with GFP‐SYT11 and Myc‐VHL. Immunoblotting after IP with anti‐GFP or anti‐Myc revealed binding between SYT11 and VHL (Figure 3A). Additionally, VHL knockdown resulted in a reduction of SYT11 ubiquitination (Figure 3B), indicating that VHL mediates the ubiquitination of SYT11. Next, we predicted the potential ubiquitination sites of SYT11 using iUbiq‐Lys (http://www.jci‐bioinfo.cn/iUbiq‐Lys) and mutated the Lys‐74 and Lys‐217 residues. In MKN1 cells, mutating both Lys sites restored SYT11 expression reduced by VHL (Figure 3C, lanes 7 and 8), whereas mutating each residue individually did not (Figure 3C, lanes 3 to 6). These findings demonstrate that VHL binds to SYT11 and promotes its degradation through ubiquitination at the Lys‐74 and Lys‐271 residues.

### 3.4 SYT11 as Well as VHL regulates SPINK1 Expression in Gastric Cancer Cells

**FIGURE 4:**
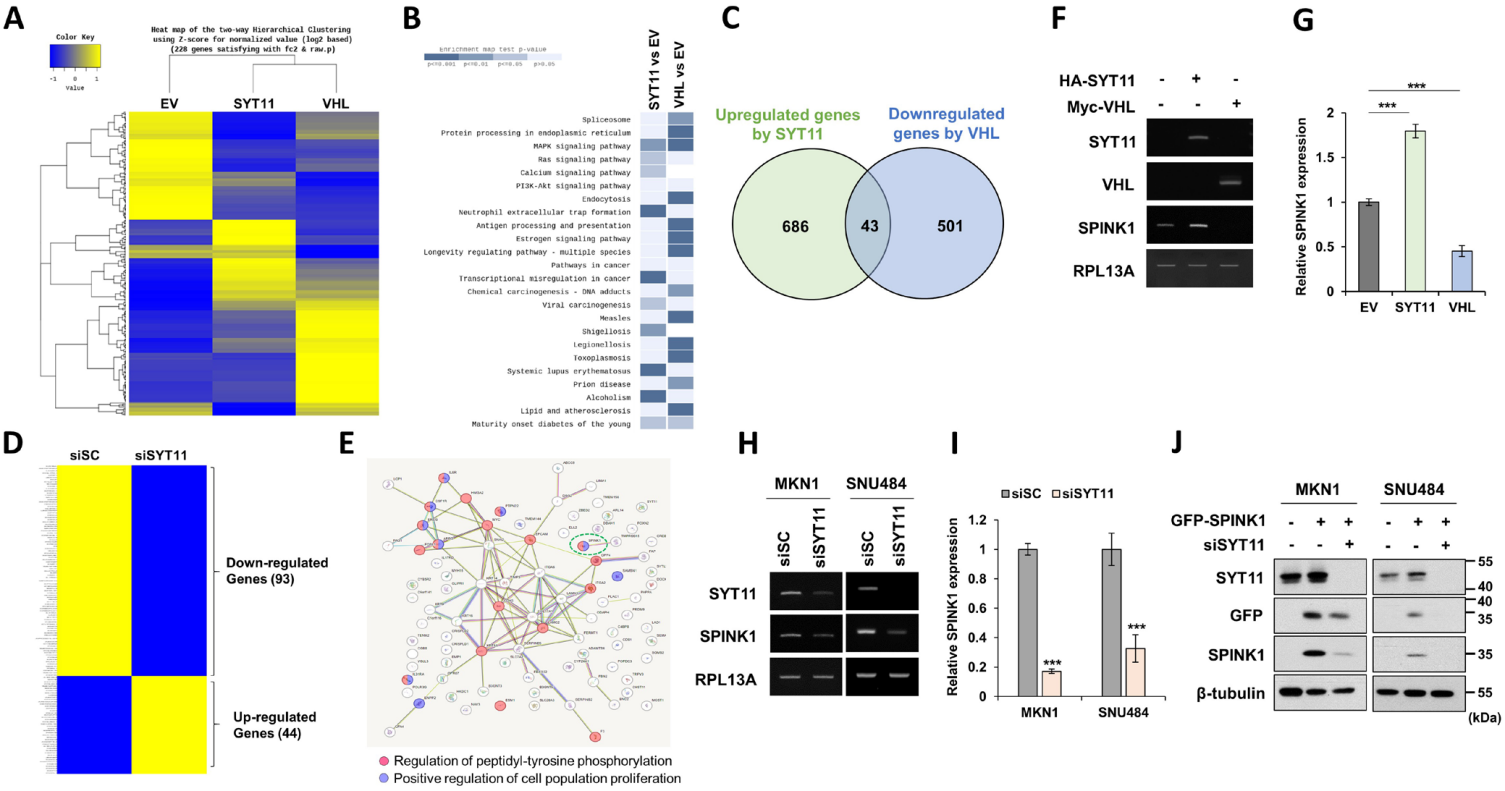
SYT11 upregulates SPINK1 expression. (A) Transcriptome sequencing analysis showing hierarchical clustering of 228 differentially expressed genes (DEGs) in MKN1 cells transfected with HA‐SYT11 and Myc‐VHL (fold change (fc) ≥ 2, exactTest raw *p* < 0.05). (B) KEGG pathway analysis. (C) Venn diagram: Overlap of SYT11‐upregulated and VHL‐downregulated genes (fc ≥ 1.5 or fc ≤ −1.5). (D) Transcriptome sequencing analysis of MKN1 cells transfected with siScramble (siSC) and siSYT11. (E) STRING analysis of 93 genes downregulated by siSYT11 treatment (fc ≤ −1.5). (F, G) RT‐PCR and qRT‐PCR analysis of MKN1 cells transfected with HA‐SYT11 and Myc‐VHL. The graph represents the SPINK1 mRNA expression analysed by qRT‐PCR. (H, I) RT‐PCR and qRT‐PCR analysis of MKN1 or SNU484 cells transfected with 40 μM siSYT11 using Lipofectamine RNAiMAX for 48 h. The graph represents the SPINK1 mRNA expression analysed by qRT‐PCR. (J) MKN1 or SNU484 cells were transfected with GFP‐SPINK1, followed by treatment with siSYT11. ***p ≤ 0.001 (Student's *t*‐test).

We performed transcriptome analysis to identify target genes regulated by VHL and SYT11 (Figure 4A,B). In MKN1 cells, 43 genes were upregulated by SYT11 overexpression and downregulated by VHL overexpression (Figure 4C). Similarly, transcriptome analysis of siSYT11‐transfected MKN1 cells revealed 93 genes downregulated upon SYT11 knockdown (Figure 4D). Notably, SPINK1 was identified as a common target in both analyses. Gene ontology analysis indicates that SPINK1 is involved in pathways related to regulation of peptidyl‐tyrosine phosphorylation and positive regulation of cell population proliferation (Figure 4E). RT‐PCR validation confirmed reduced SPINK1 mRNA expression in MKN1 cells overexpressing VHL and in MKN1 and SNU484 cells treated with siSYT11 (Figure 4F–I). SPINK1 protein expression was inhibited by siSYT11 treatment in MKN1 and SNU484 cells transfected with GFP‐SPINK1 (Figure 4J). These results suggest that VHL mediates the degradation of SYT11 and inhibits SPINK1 expression.

### 3.5 VHL Regulates the Growth and Invasion of Gastric Cancer Cells Through Downregulation of SYT11 and SPINK1 Expression

**FIGURE 5:**
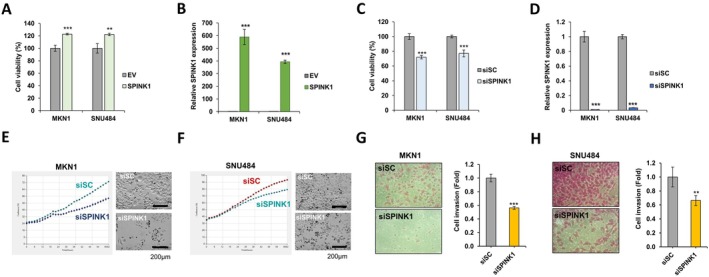
SPINK1 regulates the growth and invasion of gastric cancer cells. (A, B) Cell viability by SRB assay (A) and qRT‐PCR analysis of SPINK1 expression (B) in MKN1 or SNU484 cells transfected with GFP‐SPINK1 for 48 h. (C, D) Cell viability by SRB assay (C) and qRT‐PCR analysis of SPINK1 expression (D) in MKN1 or SNU484 cells transfected with 40 μM siSPINK1 for 48 h. (E, F) Measurement of cell proliferation by live cell imaging analysis of MKN1 or SNU484 cells transfected with siSPINK1. (G, H) Cell invasion assay in MKN1 or SNU484 cells transfected with siSPINK1. The graph shows the mean value from three independent experiments. ***p ≤ 0.001; **p ≤ 0.01 (Student's *t*‐test).

To investigate the role of SPINK1 in gastric cancer cells, we assessed gastric cancer cell growth following SPINK1 overexpression (Figure 5A,B). SRB analysis, which measures cell viability, revealed that overexpression of SPINK1 increased gastric cancer cell growth. In contrast, the growth of MKN1 and SNU484 cells treated with siSPINK1 was reduced (Figure 5C,D). Live cell imaging further confirmed the inhibitory effects of SPINK1 on the growth of MKN1 and SNU484 cells (Figure 5E,F). Additionally, siSPINK1‐treated cells exhibited reduced invasion compared to siScramble‐treated controls (Figure 5G,H).

**FIGURE 6:**
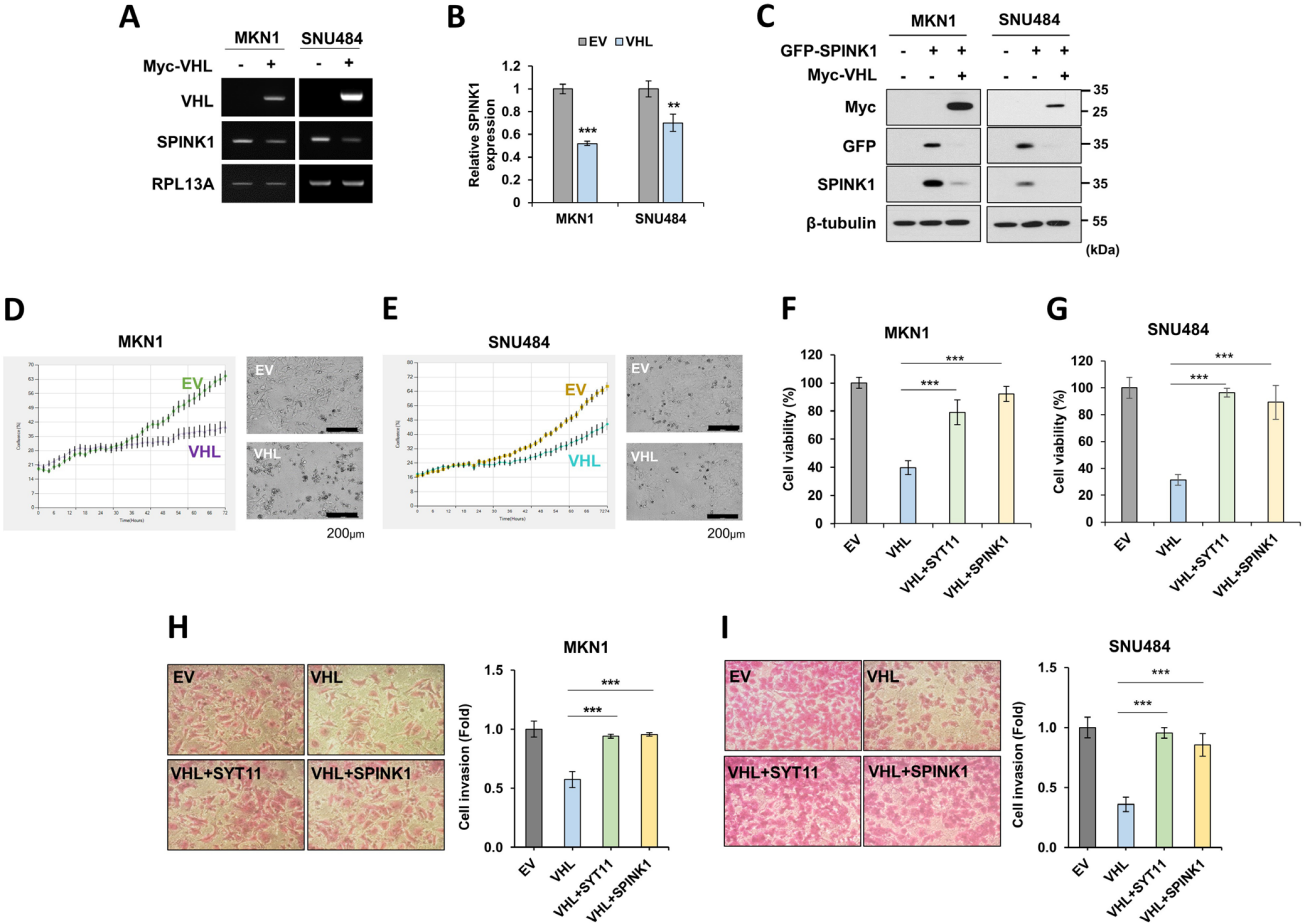
VHL regulates gastric cancer cell growth and invasion through downregulation of expression of SYT11 and SPINK1. (A, B) RT‐PCR and qRT‐PCR analysis in VHL‐transfected MKN1 or SNU484 cells. The graph represents the SPINK1 mRNA expression analysed by qRT‐PCR. (C) Western blot analysis of MKN1 or SNU484 cells transfected with GFP‐SPINK1 and Myc‐VHL. (D, E) Measurement of cell proliferation by live cell imaging analysis of MKN1 or SNU484 cells transfected with Myc‐VHL. (F, G) Measurement of cell growth by SRB assay in MKN1 or SNU484 cells transfected with Myc‐VHL, HA‐SYT11, and GFP‐SPINK1 for 48 h. (H, I) Cell invasion assay in MKN1 or SNU484 cells transfected with Myc‐VHL, HA‐SYT11, and GFP‐SPINK1. The graph shows the mean value from three independent experiments. ***p ≤ 0.001; **p ≤ 0.01 (Student's *t*‐test).

**FIGURE 7:**
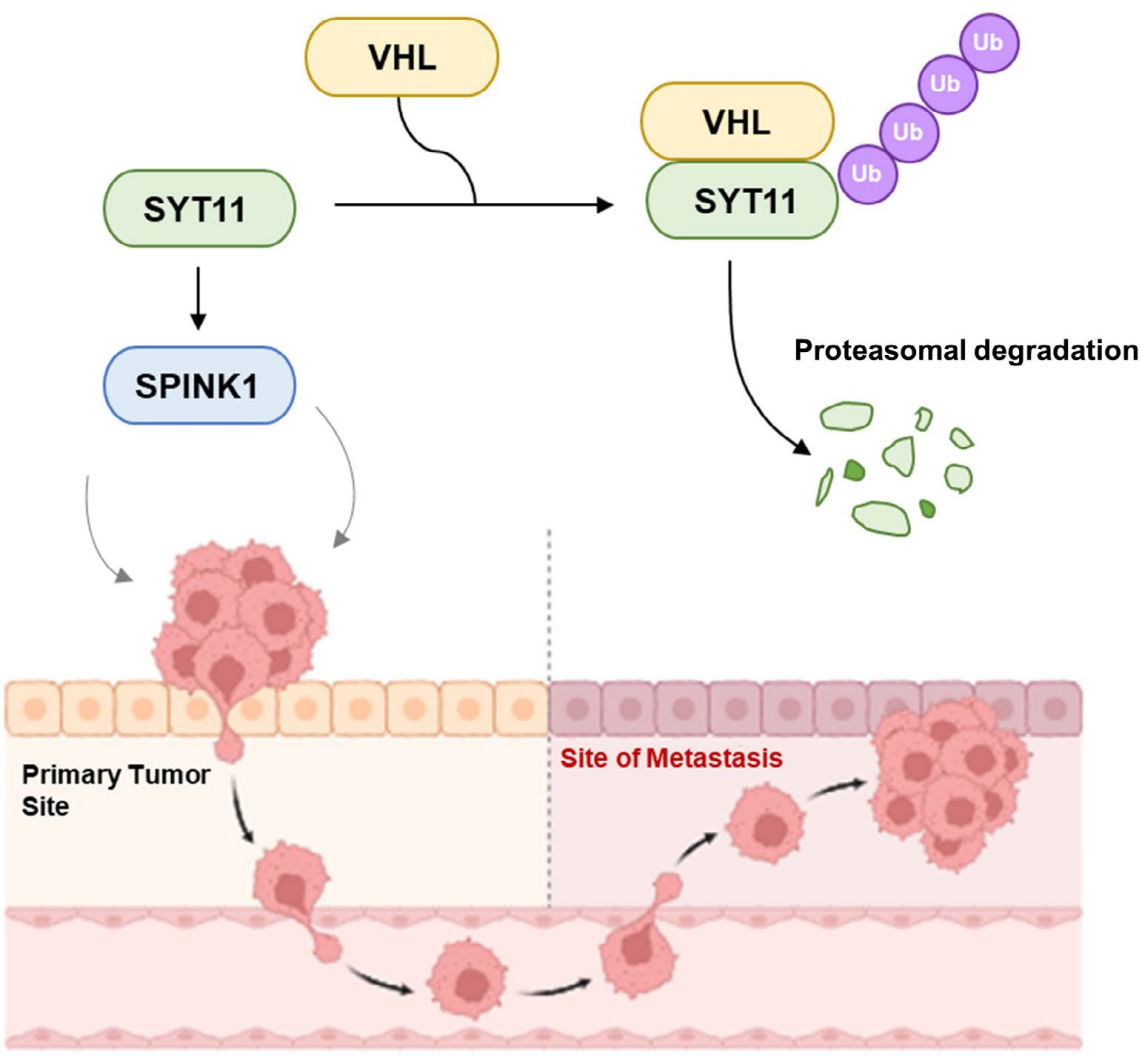
Schematic illustration: VHL degrades SYT11 through ubiquitination and inhibits the expression of SPINK1, thereby inhibiting the growth and invasion of gastric cancer cells.

Next, VHL overexpression not only decreased SPINK1 mRNA expression (Figure 6A,B) but also reduced SPINK1 protein expression (Figure 6C). Live cell imaging analysis revealed that VHL overexpression inhibited the growth of MKN1 and SNU484 cells (Figure 6D,E). Finally, we evaluated the role of SYT11 and SPINK1 in VHL‐mediated inhibition of gastric cancer cell growth. The growth inhibition of MKN1 and SNU484 cells by VHL was reversed upon overexpression of SYT11 or SPINK1 (Figure 6F,G). Similarly, the reduction in gastric cancer cell invasion caused by VHL was restored by the overexpression of SYT11 and SPINK1 (Figure 6H,I). These findings suggest that VHL inhibits gastric cancer cell growth and invasion by promoting SYT11 degradation and suppressing SPINK1 expression (Figure 7).

## 4 Discussion

Dysregulation of E3 ubiquitin ligases plays a critical role in cancer development and progression, making them promising therapeutic targets. Targeting E3 ubiquitin ligases offers a potential dual therapeutic strategy. Inhibiting the activity of these ligases to stabilise tumour suppressor proteins may help reduce cancer incidence. Additionally, regulating or eliminating the excessive expression of oncogenic proteins may contribute to the suppression of cancer progression.

Cancer treatments targeting VHL aim to mitigate the oncogenic consequences of VHL dysfunction. Current strategies under investigation include restoring the function of VHL proteins, suppression of the HIF pathway, and antiangiogenic therapy [33, 34]. The effectiveness and safety of these approaches are being evaluated in ongoing clinical trials. Further research is essential for the development of effective therapies for cancers driven by VHL dysfunction.

Although VHL is a known tumour suppressor, its role in gastric cancer remains unclear. Analyses of public data confirmed that high expression of VHL (Figure S1A) and low expression of SYT11 (Figure S1B) are positively correlated with improved prognosis of gastric cancer patients. VHL expression is negatively correlated with SYT11 expression in gastric cancer patients (Figure S1C). These analyses suggest that VHL may inhibit gastric cancer cell growth by suppressing SYT11 protein expression.

Although SYT11 has been reported as a substrate for Parkin, this is the first study to identify the role of VHL in the regulation of SYT11 protein stability. Given the low expression of Parkin and high expression of VHL in gastric cancer cell lines, we investigated the role of VHL in regulating SYT11 expression. In SYT11‐transfected gastric cancer cells, VHL reduced SYT11 protein expression (Figure 1E) and its half‐life (Figure 2A,B). We confirmed that VHL binds to SYT11 (Figure 3A), facilitating its ubiquitination‐dependent degradation, as SYT11 and ubiquitin failed to interact in the absence of VHL (Figure 3B). Furthermore, we identified the SYT11 ubiquitination site (Lys‐74 and Lys‐217) and demonstrated that mutating these two lysine residues prevented VHL‐mediated SYT11 degradation (Figure 3C).

SPINK1 is implicated in cancer progression, promoting growth and invasion in lung, colon, and pancreatic cancers [19, 20, 21, 22, 23, 24, 25]. However, its role in gastric cancer remains poorly understood. In this study, transcriptome analysis identified SPINK1 as a target gene regulated by VHL and SYT11 (Figure 4A–C). VHL overexpression (Figures 4F,G and 6A–C) or treatment with siSYT11 (Figure 4H–J) led to a reduction in SPINK1 expression, indicating that VHL reduces SYT11 expression, subsequently suppressing SPINK1, a SYT11 target. Moreover, inhibition of SPINK1 suppressed the growth and invasion of MKN1 and SNU484, gastric cancer cells (Figure 5C–H). The VHL‐induced reduction in gastric cancer cell growth and invasion was rescued by overexpression of SYT11 and SPINK1 (Figure 6F–I). Analyses of public data confirmed that high SPINK1 expression is negatively correlated with post‐progression survival of gastric cancer patients (Figure S2A), although it is not significantly correlated with overall survival (Figure S2B). These findings suggest that SYT11 may promote gastric cancer cell growth by upregulating SPINK1 expression. However, further studies are needed to fully elucidate the mechanism by which SYT11 regulates SPINK1. Unravelling the mechanisms driving SPINK1‐associated cancer progression may provide valuable insights for the development of novel therapeutic strategies. Additionally, it will be important to investigate the combined effects of HIF‐1α, a VHL target, and SYT11 on the growth, migration, and invasion of gastric cancer cells following VHL inhibition.

Research on the role of the synaptotagmin (SYT) family in cancer is rapidly advancing. Knockdown of SYTs has been shown to inhibit cancer cell proliferation, migration, and invasion [35]. SYTs have been proposed as oncogenes and prognostic markers for various cancers. Therapeutic development targeting SYTs is progressing, with an antisense oligonucleotide targeting SYT13 currently advancing to preclinical trials for gastric cancer [36]. The present study demonstrates that VHL inhibits gastric cancer cell proliferation and invasion by promoting proteasome‐dependent degradation of SYT11, thereby downregulating SPINK1. These findings provide valuable insights for developing SYT11‐targeted therapies.

## Author Contributions

**Bo‐Kyung Kim:** conceptualization (lead), formal analysis (lead), funding acquisition (lead), methodology (lead), project administration (lead), supervision (lead), writing – original draft (lead). **Ji Su Yu:** data curation (lead), formal analysis (supporting), validation (lead), visualization (lead). **Mi‐Aie Hwang:** data curation (lead), validation (lead), visualization (lead). **Misun Won:** conceptualization (supporting), writing – review and editing (lead). **Joo‐Young Im:** data curation (supporting), validation (supporting). **Dae‐Hyuk Kweon:** writing – review and editing (supporting). **Yun Gyu Park:** funding acquisition (equal), writing – original draft (equal).

## Conflicts of Interest

The authors declare no conflicts of interest.

## Supporting information


Data S1.

## Data Availability

Transcriptome sequencing dataset is available in the Korea BioData Station (K‐BDS) under registration number KAP240808.
