# Supplementary material for: VHL‐Mediated SYT11 Degradation Suppresses Gastric Cancer Cell Growth and Invasion Through Downregulation of SPINK1
Source: J Cell Mol Med. 2025 Jun 27;29(13):e70658. doi: 10.1111/jcmm.70658 (PMC12203566; doi:10.1111/jcmm.70658)
Supplement: Supplementary file 1 — Data S1. [file JCMM-29-e70658-s001.pdf]

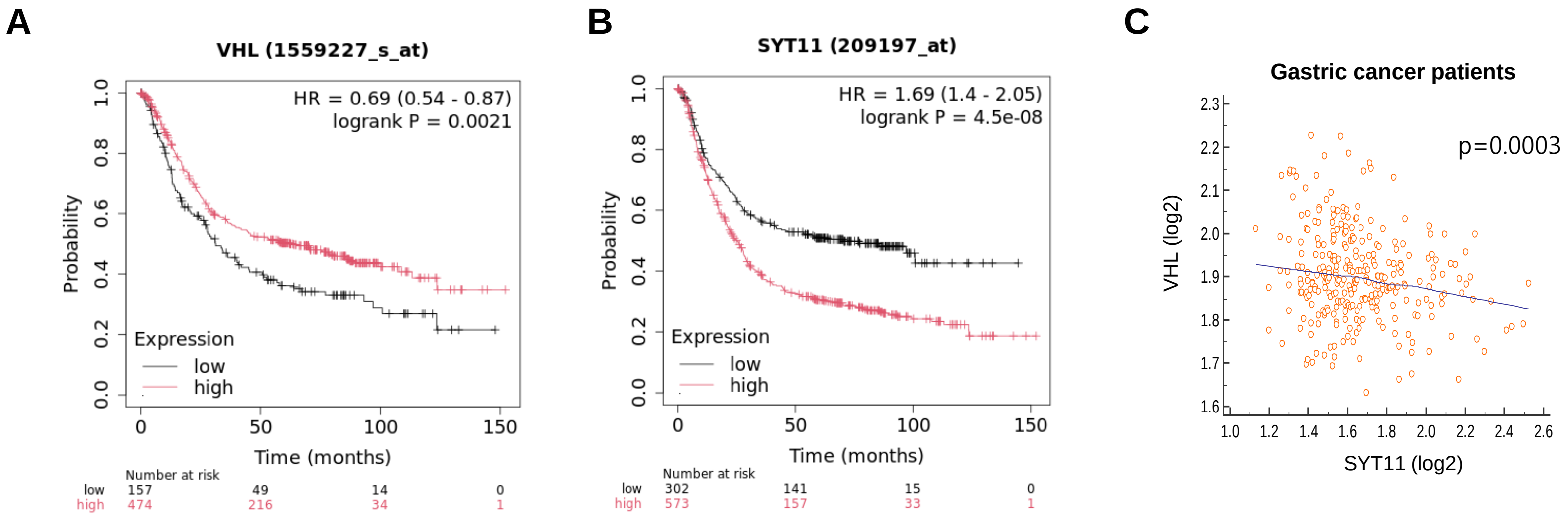

Supplementary Figure S1

A. Overall survival analysis of VHL genes using the Kaplan-Meier plotter (kmplot.com/Gastric cancer).

B. Overall survival analysis of SYT11 genes using the Kaplan-Meier plotter (kmplot.com/Gastric cancer).

C. Correlation between the expression of SYT11 and VHL in the ACRG cohort (GSE66229; n = 300).

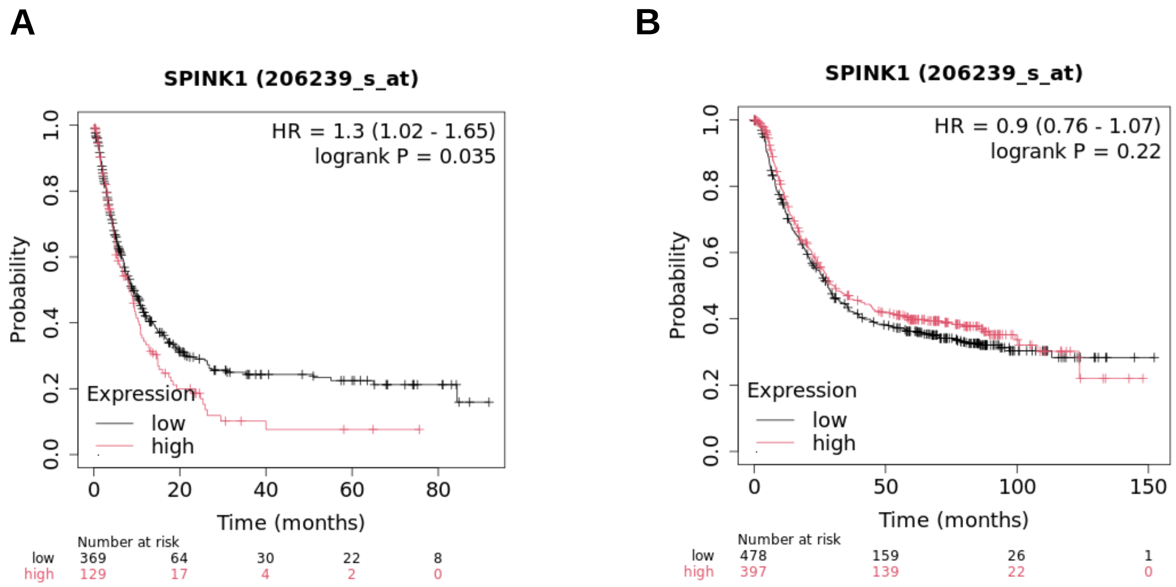

Supplementary Figure S2

A. Post-progression survival analysis of SPINK1 genes using the Kaplan-Meier plotter (kmplot.com/Gastric cancer).

B. Overall survival analysis of SPINK1 genes using the Kaplan-Meier plotter (kmplot.com/Gastric cancer).
